# Fibrous bone tissue engineering scaffolds prepared by wet spinning of PLGA

**DOI:** 10.3906/biy-1904-63

**Published:** 2019-08-05

**Authors:** Nergis ABAY AKAR, Görke GÜREL PEKÖZER, Gamze TORUN KÖSE

**Affiliations:** 1 Genetics and Bioengineering Department, Faculty of Engineering, Yeditepe University, İstanbul Turkey; 2 Molecular Biology-Genetics and Biotechnology Department, İstanbul Technical University, İstanbul Turkey

**Keywords:** Biomaterials, tissue engineering, bone, wet spinning, PLGA, rat bone marrow mesenchymal stem cells

## Abstract

Having a self-healing capacity, bone is very well known to regenerate itself without leaving a scar. However, critical size defects due to trauma, tumor, disease, or infection involve bone graft surgeries in which complication rate is relatively at high levels. Bone tissue engineering appears as an alternative for grafting. Fibrous scaffolds are useful in tissue engineering applications since they have a high surface-to-volume ratio, and adjustable, highly interconnected porosity to enhance cell adhesion, survival, migration, and proliferation. They can be produced in a wide variety of fiber sizes and organizations. Wet spinning is a convenient way to produce fibrous scaffolds with consistent fiber size and good mechanical properties. In this study, a fibrous bone tissue engineering scaffold was produced using poly(lactic-*co*-glycolic acid) (PLGA). Different concentrations (20%, 25%, and 30%) of PLGA (PLA:PGA 75:25) (Mw = 66,000-107,000) were wet spun using coagulation baths composed of different ratios (75:25, 60:40, 50:50) of isopropanol and distilled water. Scanning electron microscopy (SEM) and in vitro degradation studies were performed to characterize the fibrous PLGA scaffolds. Mesenchymal stem cells were isolated from rat bone marrow, characterized by flow cytometry and seeded onto scaffolds to determine the most appropriate fibrous structure for cell proliferation. According to the results of SEM, degradation studies and cell proliferation assay, 20% PLGA wet spun in 60:40 coagulation bath was selected as the most successful condition for the preparation of wet-spun scaffolds. Wet spinning of different concentrations of PLGA (20%, 25%, 30%) dissolved in dichloromethane using different isopropanol:distilled water ratios of coagulation baths (75:25, 60:40, 50:50) were shown in this study.

## 1. Introduction

Bone tissue engineering researches are based on the induction of the regeneration of a new functional bone by using the interactive combination of biomaterials, cells, and biosignalling molecules (Amini et al., 2012). Bone marrow-derived mesenchymal stem cells are great sources for the application of tissue engineering because of their capability to differentiate into many different kinds of cells including the osteogenic lineage (Via et al., 2012). Three-dimensional porous biomaterials, known as scaffolds, can be developed with optimal features, including strength, rate of degradation, porosity, as well as shapes and sizes (Dhandayuthapani et al., 2011). These are responsible for the promotion of cell-biomaterial interactions, cell adhesion and extracellular matrix deposition. The surface of the biomaterials should be suitable for the adhesion of the cells and should retain the cell functions. These materials are designed to supply cell proliferation. In addition, porosity is another important concern required for the transportation of gases, nutrients and regulatory factors for the survival, and proliferation and differentiation of cells. Moreover, the rate of scaffold degradation should be equivalent to the rate of tissue regeneration and they should have lack of toxicity and immunogenic response in vivo (Langer and Tirrell, 2004; Dhandayuthapani et al., 2011).Poly(lactic-*co*-glycolic acid) (PLGA) is the most well-known synthetic copolymer which consists of the monomeric units of lactide (LA) and glycolide (GA). PLGA is preferable in bone tissue engineering applications as a biomaterial to construct scaffolds for the regeneration of defected area due to its biocompatibility, biodegradability, ease of processibility, sufficient mechanical properties, and low immunogenicity and toxicity (Lu et al., 2000; Rezwan et al., 2006; Makadia and Siegel, 2011; Phua et al., 2011; Lanao et al., 2013; Azimi et al., 2014; Gentile et al., 2014). Physico-chemical features of PLGA copolymer rely on chain length, LA:GA ratio, the time that it is exposed to water, and the storage temperature. PLGA copolymer with a higher ratio of LA has exhibited the features of less hydrophilicity, resulting in less water absorption and slower degradation rate, compared to PLGA with a lower ratio of LA (Makadia and Siegel, 2011; Gentile et al., 2014; Lanao et al., 2013; Wu and Wang, 2001). The existence of methyl side groups in the structure of LA causes a higher hydrophobicity than GA. PLGA copolymer with a higher ratio of GA has also demonstrated a faster degradation rate due to the hydrophilic characteristic of GA. The rate of biodegradation is very important since it has an effect on cellular growth and regeneration of defected tissue (Lu et al., 2000; Rezwan et al., 2006; Makadia and Siegel, 2011; Phua et al., 2011; Lanao et al., 2013; Azimi et al., 2014; Gentile et al., 2014). Moreover, degree of crystallinity is an important factor due to the effects on the mechanical properties, swelling behavior, rate of hydrolysis, and biodegradation of PLGA (Makadia and Siegel, 2011; Azimi et al., 2014). Poly glycolic acid (PGA) is highly crystalline due to absence of methyl side group. If it is copolymerized with PLA, crystallinity is reduced in the form of PLGA copolymers and this situation leads to faster degradation of copolymers, compared to either PGA or PLA (Makadia and Siegel, 2011; Lanao et al., 2013; Azimi et al., 2014; Gentile et al., 2014).The wet spinning technique, a simple and inexpensive method for the preparation of PLGA monofilament fibers, is responsible for the transformation of PLGA copolymers into fibers. These are strong, elastic, and suitable fibers for tissue-engineering scaffolds. Although the electrospinning method is used more widely to get a fiber formation, wet spinning is preferable due to the higher porosity and larger pore size (Nelson et al., 2003; Neves et al., 2011; Pati et al., 2012; Tamayol et al., 2013; Azimi et al., 2014). The scaffolds fabricated with wet spinning exhibit a higher porosity than those obtained with electrospinning since fibers are deposited in a solution. Thus, cell adhesion, proliferation, and penetration into the inner part of the scaffold can be facilitated. The strong fiber structure, higher porosity and thus, easier cell adhesion and penetration render the wet spinning technique preferable for the formation of fibers from selected polymers. In this technique, PLGA copolymers can be selected due to their good processibility, which supplies the rate of degradation in a controlled manner (Nelson et al., 2003; Neves et al., 2011; Puppi et al., 2011; Pati et al., 2012; Tamayol et al., 2013; Azimi et al., 2014). In this method, once the polymer is dissolved into solution, it is passed through a spinneret and then enters into the coagulation bath solution for the formation of fibrous structure. The coagulation of the polymer occurs in another fluid that is appropriate to the spinning solvent. However, it should not be a solvent for the polymer (Gupta, 1997; Azimi et al., 2014).In this study, formation of wet-spun fibrous PLGA scaffolds using 20%, 25%, and 30% concentrations in 75:25, 60:40, 50:50 ratios of IP:DW coagulation baths were investigated for the first time in the literature for the future use in bone tissue engineering studies. Scanning electron microscopy (SEM) and in vitro degradation studies were performed for the characterization of fibrous PLGA scaffolds. The isolated rat bone marrow mesenchymal stem cells (rBMSCs) were then seeded onto selected scaffolds, and cell proliferation assay was applied to decide the most appropriate fibrous structure for cell attachment, spreading, and proliferation.

## 2. Materials and methods

### 2.1. Materials

Poly(D,L-lactide-*co*-glycolide) (PLGA), (PLA:PGA 75:25, Mw = 66,000-107,000) was purchased from Sigma-Aldrich Corporation (St. Louis, MO, USA). It was used for the fabrication of fibrous PLGA scaffolds by using dichloromethane from Merck Millipore (Burlington, MA, USA), as a solvent via the wet spinning technique. In this process, the mixture of isopropanol from Merck Millipore and distilled water (IP:DW) was also used in different ratios as a coagulation bath to obtain PLGA precipitate. In in vitro studies, alpha MEM medium (Gibco, Invitrogen, USA) supplied with 1% penicillin-streptomycin, and 10% fetal bovine serum (FBS) (Gibco, Invitrogen, USA) was used. For the characterization of isolated rBMSCs, CD29 (BD Pharmingen, USA), CD45 (BD Pharmingen, USA), CD11a (Abcam, USA), CD90 (BD Pharmingen, USA), and CD31 (Abcam, USA) antibodies were used via flow cytometry analysis. Cell proliferation on the fabricated PLGA scaffolds was analyzed with MTS reagent (Promega, USA).

### 2.2. Preparation of fibrous scaffolds

**Figure 1:**
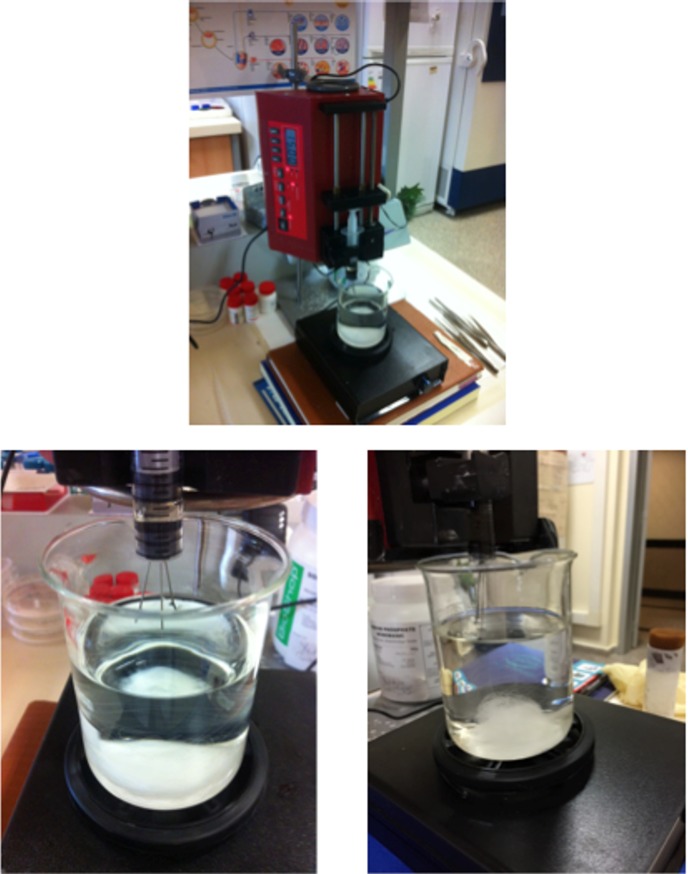
Wet spinning setup.

For the preparation of fibrous scaffolds, the wet spinning setup was designed (Figure  1). Syringe pump in vertical plane and turntable in the horizontal plane were major components of this setup. Syringe pump and turntable was responsible for the fixation of the flow rate of PLGA/dichloromethane solution in vertical plane and the rotation of the coagulation bath in the horizontal plane, respectively. In this study, syringe with 3 needles was used for the first time. Green needles with dimensions of 21 Gauge and 0.80 mm in diameter were used for wet spinning setup. The flow rate of solutions was fixed at 15 μL/min via syringe pump. The experiments were also performed at room temperature which is kept constant at 23 °C. Different concentrations of PLGA (PLA:PGA 75:25) (Mw = 66,000-107,000) in dichloromethane (20%, 25%, and 30%) were prepared and wet spun into coagulation baths of different isopropanol:dH2O ratios (IP:DW; 75:25, 60:40, 50:50). PLGA solutions were coagulated in IP:DW coagulation baths via syringe and syringe pump for the construction of the tissue engineering scaffold in the form of fibers. After wet spinning, these samples were left in coagulation bath at 4 °C overnight and then, frozen at −80 °C and freeze-dried. At the end of this process, we obtained a rigid material that cannot easily collapse. After freeze-drying, the structure became even more rigid. Then, they were cut into equal, circular pieces of 8 mm in diameter to get a clear shape/form. For in vitro studies, PLGA fibers were sterilized with 70% ethanol. Then, they were washed with PBS and dried under the laminar flow cabinet. 

### 2.3. Scanning electron microscopy

Wet-spun scaffolds prepared using different concentrations of PLGA in different coagulation bath ratios were coated with gold using Sputter Coater (Bal-tec SCD 005, Germany) and the samples were analyzed using a scanning electron microscope (Carl Zeiss EVO, Germany).

### 2.4. Degradation studies

According to the results of SEM, the most appropriate PLGA concentration and the coagulation bath ratio combinations were chosen for the best fiber formation and then, degradation studies were continued with these selected combinations. Fibers were prepared, cut into small pieces, and weighed. They were put into 5 mL of 0.09% sodium azide in phosphate buffered saline solution (PBS; 0.0095 M PO4, pH: 7.5) in sterile centrifuge tubes and they were placed into the shaking water bath at 37 °C. After 7, 15, 30, 60, 90, and 120 days of incubation, pH of PBS was measured to observe the decrease of pH values due to glycolic acid and lactic acid released by the degradation of PLGA scaffolds. After that, PLGA scaffolds were lyophilized and their weights were also measured. The rate of weight loss of PLGA scaffolds and pH change were plotted in graphs. Degraded scaffolds were also analyzed by SEM.

### 2.5. Isolation and culturing of rBMSCs

Mesenchymal stem cells (MSCs) were obtained from bone marrow of 6-7 weeks old Sprague-Dawley rats under anesthesia and aseptic conditions approved by Yeditepe University Animal Research Local Ethics Committee (YÜDHEK). Briefly, after the sacrification of rats by CO2 inhalation, the femurs and tibias were taken out and their bone marrow was flushed with cell culture medium (Alpha MEM medium supplied with 1% penicillin-streptomycin, and 10% fetal bovine serum (FBS)) into centrifuge tubes using a sterile syringe. After the extract was homogenized by pipetting, it was centrifuged at 2000 rpm for 5 min, the pellet was dissolved in culture medium and incubated in CO^2^ incubator at 37 °C, in 5% CO^2^ and 90% humidity. Medium of the rBMSCs was replenished every other day until confluency was reached.

### 2.6. Characterization of rBMSCs using flow cytometry analysis 

BMSCs were trypsinized and counted with hemacytometer when they reached the 3rdpassage. Cells (5 × 105) for each antibody and negative control were put into FACS tubes. After the addition of PBS (2 mL) into FACS tubes and centrifugation at 2000 rpm for 5 min, cell pellet was resuspended in 200 mL PBS. Then, CD29, CD45, CD11a, CD90, and CD31 antibodies were added and cells were incubated with the antibodies at 4 °C. After washing and centrifugation steps, they were resuspended in PBS and analyzed using a flow cytometer (FACSCalibur, BD Biosciences, USA). 

### 2.7. Determination of cell proliferation on the scaffolds by MTS assay 

CellTiter 96® Aqueous One Solution Cell Proliferation Assay (MTS) was used to determine rBMSCs viability on scaffolds. Selected PLGA scaffolds (20% 60:40, 25% 60:40, 25% 75:25, 30% 60:40, and 30% 75:25) were seeded with rBMSCs, and incubated in CO^2^ incubator at 37 °C, in 5% CO^2^ and 90% humidity in culture medium. The medium was refreshed twice a week. At days 7, 14, and 21, MTS reagent was added onto each scaffold and incubated in CO2 incubator for 2.5 h. Then, the absorbance values were measured for each sample at 490 nm by using ELISA plate reader (Bio-Tek, Elx800, USA). The absorbance values were transformed into cell numbers by using calibration curve constructed with MTS analysis of known cell numbers of rBMSCs.

### 2.8. Scanning electron microscope of cell seeded scaffolds

rBMSCs were seeded on wet-spun PLGA scaffolds and cultured for 20 days. The samples were fixed with 2.5% glutaraldehyde for 1 h at room temperature at days 10 and 20 of the incubation period. The scaffolds were then dried and placed onto carbon disks, coated with gold using a sputter coater (Bal-tec SCD 005, Germany) and analyzed using SEM (Carl Zeiss, EVO, Germany).

### 2.9. Statistical analysis

When evaluating the findings obtained in this study, IBM SPSS Statistics 22 for statistical analysis (SPSS IBM, Turkey) program was used. The Kruskal-Wallis test (post hoc Mann-Whitney U test) was used for the comparison of the parameters between the groups, and the Mann-Whitney U test was applied to evaluate the discrepant group. Significance was assessed at *P < 0.05 level.

## 3. Results and discussion

### 3.1. Characterization of fibrous PLGA scaffolds

#### 3.1.1. Scanning electron microscopy

**Figure 2:**
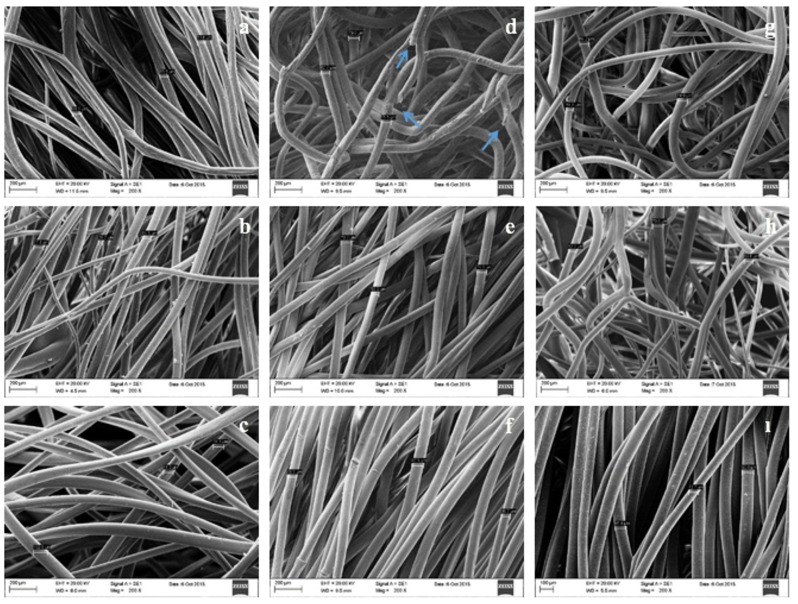
Scanning electron micrographs of wet-spun PLGA (a, b, c) 20%, (d, e, f) 25%, and (g, h, i) 30% in (a, d, g) 50:50, (b, e, h) 60:40, and (c, f, i) 75:25 (Isopropanol: dH2O) coagulation baths. 200× magnification.

After the preparation of different concentrations (20%, 25%, and 30%) of PLGA scaffolds in 75:25, 60:40, 50:50 (IP:DW) coagulation baths, SEM analysis was carried out. According to the results, PLGA solutions at concentrations of 20%, 25%, and 30% formed fibrous structures when they were spun in coagulation baths with IP:DW ratios of 50:50, 60:40, and 75:25 (Figure  2). Chosen polymer concentration, solvent, and coagulation bath compositions are responsible for the formation of fibers by wet spinning setup. Polymer flow rate from the syringe and the speed of the turntable are other parameters affecting the formation and properties of fibers. Longitudinal grooves between the fibers that were observed in SEM images were thought to supply contact guidance and to result in increased surface area which improves cell attachment (Nelson et al., 2003; Pati et al., 2012; Salamian et al., 2013). Several factors affect the scaffold morphology in wet-spinning protocol including polymer concentration, features of coagulation bath, nature of coagulants, injection rate of syringe pump, and diffusivity of components. Precipitation rate can be affected by the solubility of the polymer in coagulation bath. The larger difference in solubility parameters between the polymer and the components of coagulation bath can generally imply a faster precipitation rate. Sukitpaneenit and Chung (2009) investigated the solubility of PVDF in either water or alcohol that was used as a coagulant. N-methyl-2-pyrrolidone (NMP) was used as a solvent for the preparation of polymer solution, and this study showed that the precipitation rates follow the order: water > methanol > ethanol > IP. Addition of isopropanol to water in the coagulation bath causes a decrease in precipitation rate. Alcohol concentration in the coagulation bath can also affect the mechanical properties of the material formed. Increasing alcohol concentration can lead to slower diffusion and thus slower precipitation rates which can cause lower mechanical properties of materials (Deshmukh and Li, 1998; Zuo et al., 2006; Ali et al., 2007; Sukitpaneenit and Chung, 2009; Ahmad et al., 2012). On the other hand, too low alcohol concentration in coagulation baths can prevent precipitation and fibers cannot be formed. In our study, it was observed that coagulation baths containing less than 50% IP could not precipitate any of the polymer solutions (20%, 25%, and 30%) and fibers were not formed (Data not shown). For this reason, 50:50, 60:40, and 75:25 (IP:DW) coagulation baths were selected for this study.PLGA scaffold formation in coagulation baths with the compositions of 60:40 and 75:25 (IP: DW) were more successful in wet spinning regardless of the PLGA concentrations (Figures 2b, 2c, 2e, 2f, 2h, 2i). It was observed that continuous fibers were formed in all samples except in the 25% PLGA that was spun in 50:50 coagulation bath (Figure  2d). In this sample, breaks were observed in fiber structures, indicating that 25% PLGA concentration cannot be successfully spun in 50:50 coagulation bath. Moreover, there was a reproducibility problem in this coagulation bath. It could be due to higher DW concentration of 50:50 coagulation bath that was not miscible enough with dichloromethane which was the solvent of the PLGA copolymer. Therefore, 50:50 coagulation baths were omitted from the rest of the experiments. Since high alcohol concentration in coagulation baths cause slower diffusion (Deshmukh and Li, 1998; Ali et al., 2007) and slower precipitation of the polymer, handling and optimization of structures formed in coagulation baths with 75:25 (IP:DW) ratio were problematic. Especially in 20% PLGA concentration, there were optimization problems in 75:25 (IP:DW) coagulation bath even if the results of SEM were satisfying (Figure  2c) due to high solvent concentration. Thus, PLGA 20%-75-25 (20% PLGA wet-spun in 75:25 IP:DW coagulation bath) was not used further. Coagulation baths with 75:25 (IP:DW) ratio could be thought as a weak coagulation bath due to high alcohol concentration compared to coagulation baths with 60:40 (IP:DW) ratio. On the other hand, higher polymer concentrations such as 25% and 30% in 75:25 (IP:DW) coagulation bath led to successful fiber formation (Figures 2f and 2i). Polymer concentration is one of the factors that affect the mechanical properties of wet-spun materials. The critical polymer concentration should be determined to obtain optimum mechanical properties (Sukitpaneenit and Chung, 2009; Idris et al., 2017). Generally, increase in polymer concentration can exhibit an increase in mechanical properties (Sukitpaneenit and Chung, 2009). However, higher polymer concentration solutions beyond the critical viscosity can be usually difficult to form in fibrous structures due to the problems with the penetration of the coagulant into the polymer matrix and also, weaker mechanical properties can be detected at a polymer concentration lower than the critical viscosity (Sukitpaneenit and Chung, 2009; Bakeri et al., 2010; Idris et al., 2017).Due to the findings and considerations above, 20%-60-40 (20% PLGA wet-spun in 60:40 IP:DW coagulation bath), 25%-60-40 (25% PLGA wet-spun in 60:40 IP:DW coagulation bath), 30%-60-40 (30% PLGA wet-spun in 60:40 IP:DW coagulation bath), 30%-75-25 (30% PLGA wet-spun in 75:25 IP:DW coagulation bath) and 25%-75-25 (25% PLGA wet-spun in 75:25 IP:DW coagulation bath) samples were chosen for the further usage. 

#### 3.1.2. Degradation studies and scanning electron microscopy of PLGA scaffolds

##### 3.1.2.1. Degradation studies

**Figure 3:**
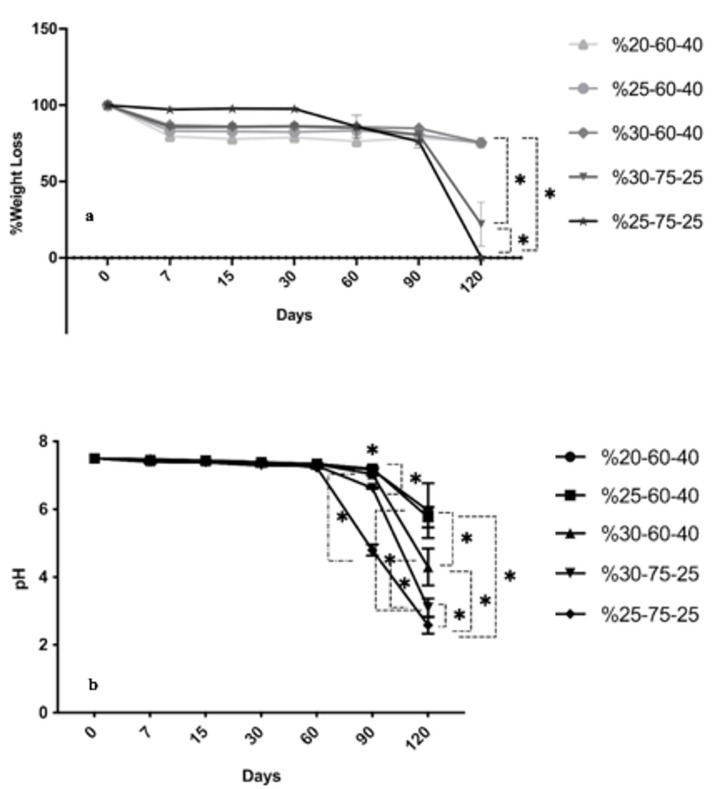
The rate of (a) weight loss and (b) pH decreasing as a function of time (days) (*P < 0.05) during biodegradation of fibrous PLGA scaffold.

Lactic acid:glycolic acid (LA:GA) ratio of PLGA affects the crystallinity of copolymer and changes the mechanical properties and physico-chemical properties such as hydrophilicity, swelling behavior and thus, degradation rate. PLGA (75:25 LA:GA ratio) with higher amount of LA shows less hydrophilicity that causes less absorption of water and thus, slower degradation. PGA is known as highly crystalline. When it is copolymerized with PLA which has relatively low crystallinity, the resulting copolymer, PLGA, becomes moderately crystalline and its hydration and hydrolysis properties are changed. Thus, faster degradation of copolymer is obtained compared to either PGA or PLA (Makadia and Siegel, 2011; Lanao et al., 2013; Azimi et al., 2014; Gentile et al., 2014). If the diffusion of water into a material is not faster than the degradation of the polymer, it ends up with the hydrolysis of bonds on the polymer surface. On the other hand, if the degradation of polymer is slower than the diffusion of water into the polymer, bulk erosion of the polymer occurs (Burkersroda et al., 2002).In this study, wet-spun PLGA scaffolds were kept in PBS during 120 days in order to evaluate the degradation behavior of the samples. It was observed that scaffolds maintained at least 80% of their weight during the first 90 days of the incubation period, regardless of concentration and coagulation bath composition (Figure  3a). However, an increase in the degradation rate of PLGA scaffolds was observed after day 90, especially in 30%-75-25 and 25%-75-25 samples. The 30%-75-25 lost 80% of its weight while 25%-75-25 completely degraded after 120 days of incubation. This was possibly due to the slower surface erosion in the first 90 days of incubation followed by a bulk erosion. At day 120, the weight loss of 25%-75-25 was significantly higher than the other groups (*P < 0.05). The weight loss of 30%-75-25 was also found significantly higher than the groups of 20%-60-40, 25%-60-40, and 30%-60-40 (*P < 0.05). During the degradation of PLGA, pH decrease can be seen due to the release of lactic acid and glycolic acid residues (Holy et al., 1999; Zolnik and Burgess, 2007). A major drop in pH is undesirable because it can cause necrosis in cells and tissues. Besides, degradation of PLGA fibers did not cause too much pH change in the first 90 days except for 25%-75-25 sample (Figure  3b). Results were parallel to the obtained weight loss data. It was due to the adequate buffering capacity of PBS that was used as degradation medium for the samples, when the degradation was in the form of surface erosion. Buffer systems in the blood perform the same task in vivo with better performance compared to PBS, preventing a rapid pH change due to degradation (Verma et al., 2010). At day 90, pH level of 25%-75-25 sample was found significantly lower than those of all the other groups (*P < 0.05). Moreover, a significant pH decrease was observed in 30%-75-25 compared to %20-60-40, %25-60-40 and %30-60-40 (*P < 0.05). pH of 30%-60-40 was significantly lower than those of the samples of 20%-60-40 and 25%-60-40 (*P < 0.05). At day 120, the pH level of 25%-75-25 was significantly lower than those of the other groups (*P < 0.05). The 30%-75-25 also showed lower pH values than 20%-60-40, 25%-60-40, and 30%-60-40 (*P < 0.05) at day 120. Moreover, pH levels of 20%-60-40 and 25%-60-40 were significantly higher than that of 30%-60-40 (*P < 0.05). The results of degradation analysis of wet-spun PLGA scaffolds showed that the samples obtained in 60:40 coagulation baths degrade slower, which renders them preferable for bone tissue engineering applications.

##### 3.1.2.2. Scanning electron microscopy of PLGA scaffolds

**Figure 4:**
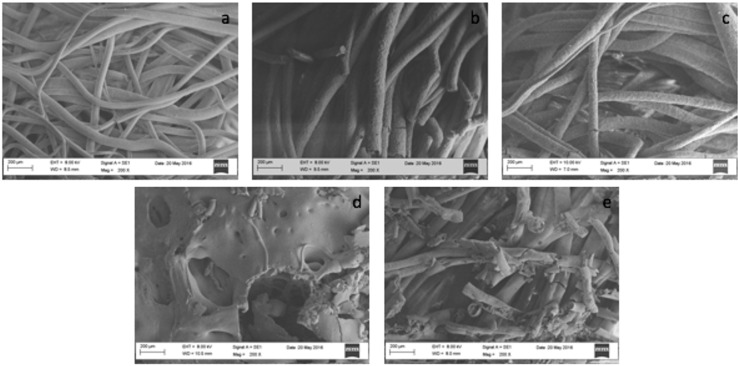
Scanning electron micrographs of wet-spun PLGA at the end of 90 days of incubation period; a) 20%-60-40, b) 25%-60-40, c) 30%-60-40, d) 25%-75-25, e) 30%-75-25.

The degraded PLGA scaffolds were also examined by SEM in order to observe the extent of deterioration in the structures. Analyses were performed at the end of the 90-day period at which the degree of degradation could be observed most clearly. In SEM images of degraded samples, it was observed that the deformation of fibrous structures in samples that were spun in 75:25 coagulation bath was more dramatic (Figure  4). During the 90 days of the incubation period, the fibers underwent erosion and some of the fibers were broken. After 60 and 90 days of incubations, decreases in pH values were observed for 25%-75-25 and 30%-75-25, respectively, which possibly further increased the degradation rate. As a result of both degradation and SEM analyses of degraded samples, the 20%-60-40 sample was determined as the one having the lowest degradation rate.

### 3.2. In vitro cell-material interactions

#### 3.2.1. Characterization of isolated rBMSCs using flow cytometry analysis

**Figure 5:**
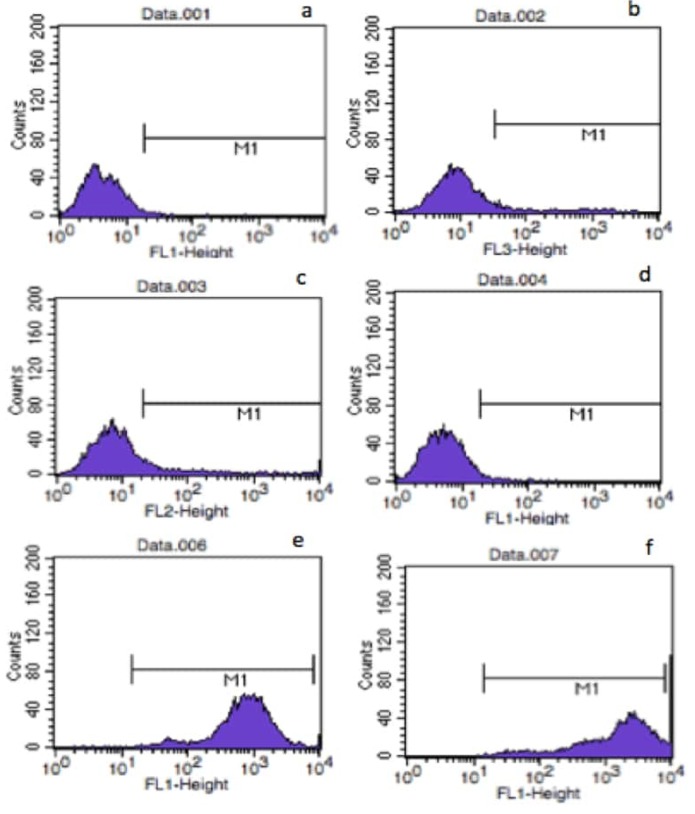
Flow cytometry histograms of rBMSCs a) Negative control; labeled with (b) CD45, (c) CD11A, (d) CD31, (e) CD29, (f) CD90 antibodies.

Since the objective of this study is to obtain a fibrous scaffold for bone tissue engineering applications, it is essential to assess interactions of cells with the obtained scaffolds. For this purpose, rBMSCs were isolated and characterized using flow cytometry. Cell surface antigen profiles of rBMSCs were investigated to analyze their stem cell properties. The results of flow cytometry analysis demonstrated that the cells were positive for mesenchymal stem cell surface antigens CD29 (99.55%) and CD90 (94.67%). They had low expression for hematopoietic stem cell markers CD11A (9.74%) and CD45 (5.17%), and were also negative for an endothelial cell surface marker CD31 (2.38%) (Figure  5) showing that the isolated cells have cell surface antigen profile of mesenchymal stem cells.

#### 3.2.2. Determination of cell proliferation by MTS assay 

**Figure 6:**
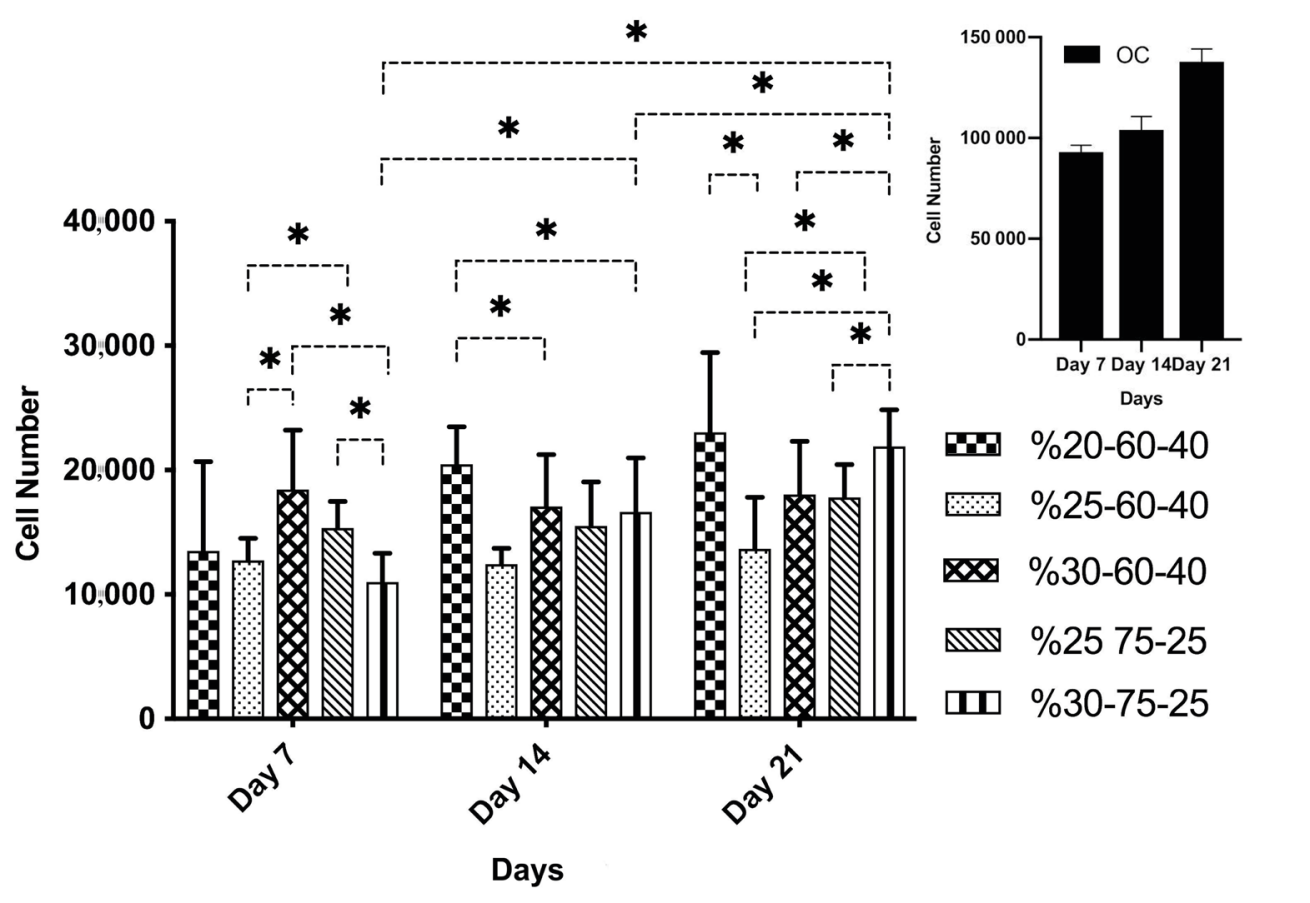
Cell proliferation on different PLGA scaffolds (20%-60-40, 25%-60-40, 25%-75-25, 30%-60-40, and 30%-75-25) after 7, 14, and 21 days of incubation. Initial cell seeding was 2 × 104 cells/sample (*P < 0.05). Indent on the top right corner shows the cell proliferation on Tissue Culture Plate and OC stands for the cells seeded into wells of the tissue culture plate (only cell).

In order to obtain an appropriate scaffold for bone tissue engineering, an adequate cell adhesion and proliferation of adhered cells are necessary to populate the scaffold. CellTiter 96**®** AQueous One Solution Cell Proliferation Assay (MTS) was applied to test the adherence and proliferation of rBMSCs on selected PLGA scaffolds (20%-60-40, 25%-60-40, 30%-60-40, 30%-75-25, 25%-75-25). It was demonstrated by the MTS assay that the seeded cells were attached to all selected wet-spun scaffolds, but higher cell proliferation was observed on the samples of 20%-60-40 and 30%-75-25 than that of the others throughout 21 days of incubation (Figure  6). At day 7, the cell number on the sample of 30%-75-25 and %25-60-40 was significantly lower than that of both 25%-75-25 and 30%-60-40 (*P < 0.05). At day 14, cell proliferation on the samples of 20%-60-40 was significantly higher than the samples of 30%-60-40 and 30%-75-25 (*P < 0.05). At day 21, cell proliferation on 30%-75-25 samples was also significantly higher than that of the 25%-60-40, 25%-75-25, and 30%-60-40 (*P < 0.05). Moreover, the cell number on the sample of 25%-60-40 was significantly lower than that of 20%-60-40, 25%-75-25 (*P < 0.05). Moreover, in the group of 30%-75-25; there was a statistically significant difference in cell proliferation (*P < 0.05) between the 7, 14, 21 days of incubation. The amount of cells at day 7 was significantly lower than that at days 14 and 21 (*P < 0.05) and the cell number at day 14 was significantly lower than that at day 21 (*P < 0.05). As a result, the 20%-60-40 sample was selected as the best sample in terms of cell adherence and proliferation according to MTS cell proliferation assay and degradation analysis since the samples with a 30% PLGA concentration had rapid degradation according to the degradation analysis. The sample of 20%-60-40 was also easier to prepare and handle.

#### 3.2.3. Scanning electron microscopy of cell seeded scaffolds

**Figure 7:**
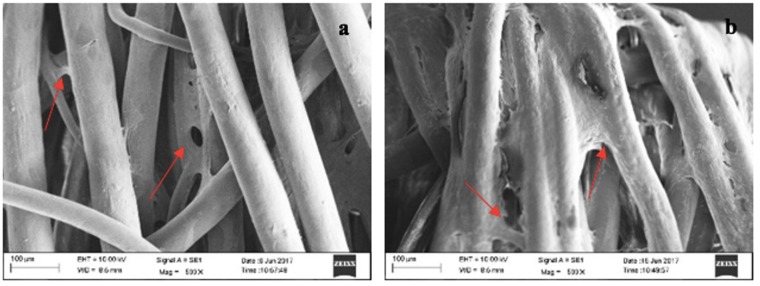
Scanning electron micrographs of MSCs seeded onto the PLGA fibers (20%-60-40) at day 10 (a) and 20 (b) with 200**×** magnification.

Cell-seeded 20%-60-40 PLGA scaffolds were examined in SEM to evaluate cell adhesion. At day 10, cell spreading and proliferation were observed on this scaffold (Figure  7), and the surfaces of the scaffolds were completely covered by the seeded cells at day 20. Wet-spun fibrous scaffolds obtained in this study were favorable for cells since fibers could supply large surface area for the cells to adhere and high porosity to ensure cell migration and diffusion of O2 and nutrients through the interiors for cells to populate the scaffold evenly (Pati et al., 2012). Having an appropriate pore size or porosity is an advantageous situation since it leads to the penetration of cells inside the scaffolds and the efficient flow of O2 and nutrients for efficient cell growth and cell spreading (Abay et al., 2016).

## 4. Conclusion

Critical size bone defects generally fail to be healed without intervention and require reconstruction surgeries. Conventional methods such as grafting have high risks of infection and immune rejection. Tissue engineering methods have the capability for the combination of cells, scaffolds, and signaling molecules to solve the problems associated with the healing of those defects. Scaffolds serve as a substitute for damaged tissues by providing the mechanical support until regeneration of healthy tissue is completed and act as a template for the guided organization of cells.In this study, different concentrations of PLGA were wet spun in different coagulation bath combinations to fabricate fibrous tissue engineering scaffolds. They were seeded with rBMSCs to observe cell adhesion and proliferation which are essential for tissue engineering. The 20% PLGA wet spun in 60:40 (IP:DW) coagulation bath was selected as the most appropriate one for bone tissue engineering in terms of fiber structure, degradation behavior and cell proliferation. In order to obtain a bone tissue using the scaffolds fabricated in this study, more extensive in vitro and in vivo experiments are required.

## Acknowledgements

This study was funded by TÜBİTAK (The Scientific and Technological Research Council of Turkey) (Grant No: 114S556).
